# Enhancing research quality through defined and consistent terminology

**DOI:** 10.1016/j.apjon.2025.100659

**Published:** 2025-01-27

**Authors:** Negin Dorri

**Affiliations:** Department of Psychology and Educational Sciences, University of Tehran, Tehran, Iran

## Introduction

A critical element of high-quality scientific research lies in its well-defined and consistent terminology. The art of clear writing ensures that readers easily understand and follow the content of the study. In a field as impactful as oncology nursing, where research outcomes influence clinical practices and patient care, precise and clear writing is essential. The impact of oncology nursing's findings extends beyond its domain, benefiting health care providers, policymakers, patients, and family caregivers. The diversity in audience and scope highlights the importance of using consistent and well-defined terminology to foster communication, promote collaboration, and avoid misunderstandings. This editorial elaborates on the significance of defining key terms in articles and guides achieving clarity and consistency in research communication, especially for early-career scholars.

## Titles and abstracts

The title and abstract, as the first elements readers encounter, play a crucial role in shaping their expectations and ensuring accessibility for both specialists and non-specialists. These components guide readers' initial understanding and set the tone for the research. A well-crafted and engaging title highlighting the study's primary focus can appeal to a broad audience. Similarly, by providing a concise summary of the purpose and key findings, the abstract ensures alignment with the manuscript's content and consistency throughout.

## Keywords

Keywords are crucial in guiding the readers, reinforcing the content's purpose, and categorizing the article. They enhance an article's discoverability, relevance, and integration into broader academic discussions. Many platforms have been designed to standardize the terms and concepts used in the scientific literature and improve the retrieval of relevant information. Medical Subject Headings (MeSH) is an example of a highly recognized platform that aligns articles with universally recognized terminologies and increases academic visibility.^1^ However, many early-career scholars neglect this crucial step, submitting manuscripts without aligning their keywords with MeSH or similar databases. This omission can limit the article's accessibility, reduce interdisciplinary engagement, and hinder its integration into the broader scientific dialogue.

## Consistency

Ensuring clarity in writing profoundly depends on applying consistent terminology. Once keywords are selected, it is the writer's responsibility to consistently use them throughout the manuscript. This responsibility is key in maintaining uniformity, avoiding scientific synonyms or alternative technical expressions that could confuse or distract the reader's attention. For instance, if “treatment outcome” is chosen as a keyword, writers must ensure its consistent use as the primary term throughout the literature rather than switching to alternatives like “clinical efficacy” or “patient-relevant outcomes.” Consistent terminology ensures the content is easily understood without requiring the readers to seek external references for definitions.

## Defining the key terms

Defining scientific key terms is not just about offering precise explanations for specialized concepts or phrases used in the study. It is also about providing a learning opportunity. Clear definitions establish a foundation for deeper exploration of key concepts, ensuring alignment between the scholars' intended use of terms and the readers' understanding. Ambiguous definitions may lead to varied interpretations, highlighting the importance of not presuming readers’ familiarity with specific terminology. The term “cancer survivor,” as discussed in the case study, exemplifies why precise definitions are vital for ensuring accurate and consistent interpretation.

### Case study: Cancer survivorship

Relying on a basic understanding of terms without having a comprehensive understanding of their in-depth meaning has become an unconscious practice, as reflected in how societies frequently use these terms daily. The National Cancer Institute (NCI) defines a cancer survivor as any individual diagnosed with cancer, starting from the time of diagnosis and continuing throughout their lifetime.^2^ This broad definition includes individuals undergoing active treatment, living with cancer, or remission. However, the term is often misunderstood by those unfamiliar with oncology-specific terminology. Many interpret it as applying solely to individuals who have completed treatment and are in remission, often described as having “no evidence of disease” or being “cancer-free,” which implies the absence of residual cancer and no chance of recurrence. This misunderstanding can lead to miscommunication and misinterpretation of research findings. Providing clear and precise definitions is vital to bridge this gap and ensure better understanding for early educators and non-healthcare audiences.

Furthermore, in studies with multiple definitions of the same key term, specifying which definition or theoretical framework guides the work is highly recommended. This approach provides readers with clarity on the context and actively engages them in interpreting the findings accurately. For instance, when presenting the NCI's definition of a cancer survivor alongside the American Cancer Society's definition, which also emphasizes inclusivity from the time of diagnosis across all stages of the disease,^3^ researchers should explicitly state which definition frames their study regardless of their similarity. This practice enhances clinical relevance and ensures greater precision for academic and practical applications.

## Cultural diversity and global context in definitions

Defining keywords also requires thoughtful consideration of cultural differences, as interpretations can differ significantly across regions and contexts. Cultural differences highly influence how terms are perceived, making it essential for researchers to consider these variations to ensure their work remains relevant to local, national, and global audiences. Providing culturally aware definitions promotes mutual understanding across health care systems and societal contexts. Authors can achieve this by offering context-specific explanations or acknowledging cultural differences in interpretation while specifying the definition guiding their work. Addressing cultural diversity ensures that health care terminology remains inclusive and applicable across various regions.

### Case study: Palliative care and hospice care

The distinction between “palliative care” and “hospice care” underscores the crucial role of precise definitions, in health-related research. This is especially important in understanding and applying these terms in the context of cultural differences. Palliative care, a comprehensive approach that addresses physical, emotional, and psychosocial needs, is designed to enhance the quality of life for individuals with life-limiting illnesses, regardless of their treatment status. In contrast, hospice care, a subset of palliative care, offers care focused on comfort for patients with a prognosis of six months or less, prioritizing supportive measures over curative treatments.^4^

Hospice care and palliative care are frequently conflated in many countries, especially where hospice care is not formally recognized as a distinct concept. This lack of recognition is often due to socioeconomic barriers, such as budget constraints, limited health care resources, and perceived stigma stemming from fear of judgment, resulting in inadequate resources for its implementation. Even in countries where hospice care is available, it may be limited to hospital-based services or vary significantly in scope. These inconsistencies can create misunderstandings in both practice and research. The key is to strike a balance between cultural norms and global standardization through precise terminology, ensuring the research's universal applicability.

## Recommendations and discussion

Clear and consistent scientific writing relies on deliberate strategies to refine terminology and bridge understanding across diverse audiences. Table 1 presents the descriptions and some intended outcomes of the editorial's recommendations that early-career researchers can implement, based on journal guidance, to improve the accessibility and impact of their studies.

**Table 1:** Terminological consistency in scientific writing.

	Recommendation	Description	Outcomes
1	Selection of keywords	Choosing key terms from standardized sources such as MeSH.	- Enhanced search accuracy - Improved indexing & discoverability - Ease of navigation - Supporting advanced search
2	Explicit definition	Defining terms using authoritative sources or theoretical frameworks and providing a structured and comprehensive understanding of the term's context and usage.	- Better understanding - Discipline-specific focus - Efficiency - Global relevance - Clarity
3	Adaptation	Considering cultural, regional, and universal distinctions when defining terms.	- Enhanced understanding - Improved engagement - Knowledge sharing - Acknowledging cultural diversity - Universal applicability
4	Consistency in usage	Using chosen scientific terms consistently throughout the manuscript.	- Clarity - Harmonization
5	Addition of glossaries	Include a glossary for technical terms if the journal permits.	- Facilitates learning - References for future use
6	Feedback & Peer review	Obtaining feedback from collaborators and applying peer review's comments.	- Enhanced credibility - Refinement of ideas - Adherence to standards - Improved quality and accuracy

MeSH, Medical Subject Headings.

By adopting practices such as selecting standardized keywords, offering explicit definitions, and ensuring consistent usage, researchers improve the clarity, accessibility, and relevance of their work. These measures make research more understandable and enable readers to interact with and implement the findings more effectively.

## Conclusions

Promoting terminological clarity will remain essential as integrated approaches to care continue to expand. Researchers, educators, and practitioners must work together to ensure their contributions are thorough, inclusive, and relevant to the diverse perspectives of readers from various academic, cultural, and educational contexts. This coherence facilitates the advancement of knowledge and its practical application, ensuring that the study has a tangible impact on real-world settings. This harmony encourages shared understanding and enhances the utility of research in real-world settings.

## Funding

This study received no external funding.

## Declaration of competing interest

The author declares no conflict of interest.

## Declaration of generative AI and AI-assisted technologies in the writing process

During the preparation of this work, the author used Grammarly software to check for grammatical accuracy and presentation. After using this tool, the author reviewed and edited the content as needed and takes full responsibility for the content of the publication.
